# Dermatological Notes

**Published:** 1893-03

**Authors:** Isadore Dyer

**Affiliations:** Tulane University, New Orleans, La.


### DERMATOLOGICAL NOTES.

BY ISADORE DYER, M. D.,
Tulane University, New Orleans, La.

I. LEPROSY IN BRITTANY, FRANCE.—Quite a startling report of extensive leprosy in Brittany, is made by Dr. Zambaco-Pacha, in the December number of the *Annales de Dermatologie et Syphilographic.* Many of the cases reported closely resemble what has heretofore been described as Woman's disease. Dr. Z. logically investigated the cases and reports them in detail; presenting photographs of the important cases. It is thought that leprosy had disappeared in Central Europe since the 16th century, only isolated cases being reported. Concluding a long and interesting paper Dr. Z. summarizes as follows:

1. Leprosy exists in Brittany, and it is the new disease (Syringo-myelitis) belived to have been discovered. The *Analgesic Whitlow* is nothing but the classic *Lepra mutilans.* In addition we have met the anesthetic form of Daniellssen—the claw hand, with muscle atrophy; loss or interference with sensation, etc.; the ulcerating or *lazarine* form, and even the tubercular form.

2. Besides these classic types of leprosy there are (and by far the greater number, fortunately) those forms which are attenuated, light or modified. The transitions however, are established. Cases occurring in all grades, separated only by slight lines from one another.

3. Most of the cases considered affected by the new disease, called *syringo-myelitis*, are only lepers, attacked by the anesthetic type of Daniellssen, but somewhat attenuated or slightly modified.

4. Finally, leprosy, like scrofulosis, consists of a large class of affections, among which may be included many other maladies to-day considered new and distinct diseases. Included among these are *sclerodern* and those indefinite conditions, which are designated *morphea*, of which many preserve their sensibility.

But the absence of only one symptom scarcely justifies the creation of a new disease. There are true *lepromes*, which have sensation. In those affected with *morphea*, there are spots which are anesthetic. There is no reason then to separate these cases from the generic class of leprosy. All the more is one led to isolate a type of *esthetic* leprosy as distinct from the anesthetic type.

---

II. Etiology of Psoriasis.—Dr. Ludwig Nielssen, in the *Monatshefte fur Praktische Dermatologie*, (Vol. 15, Nos. 7 and 8), contributes an article on psoriasis, the result of observation in over one thousand cases. In the treatment of the disease Dr. Nielssen prefers iodides to asenic. The disease occurred as two to one in males with females. Heredity, he concludes, is a negative or uncertain factor. Rheumatism appeared largely associated. Acute diseases seemed to hasten the recovery. Diatheses, constitutional conditions, blood infection, and the neuopathic theory, were all rejected as lacking proof. The parasitic origin of the disease seemed to carry more evidence. Dr. Nielssen compares psoriasis to *pityriasis versicolor*, a parasitic disease, rarely contagious, having predilection for certain individuals, tending to recur and having a characteristic fungus. In psoriasis parasiticides act favorably. Fungi are found in the lesions and they probably belong to the *hyphomycetic* or the *schizomycetic* group.

III. Pityriasis Rosea.—Dr. Lasaar, of Berlin, from his investigation pronounces *pityriasis rosea* and scaling ringworm of the body as identical. His recent communication to the Berlin Society of Dermatology brings out some interesting points in the etiology of *pityriasis rosea*. Lasaar has observed the eruption appearing on young women beneath new knitted undershirts, not previously washed. He also noticed the disease under new washed linen garments, worn for the first time. A similar condition occurred after the wearing of linen garments which had been kept for a long time in chests or closets, thereby offering irritable soil to the parasites. For treatment Dr. Lasaar suggests solution of zinc or sugar of lead, or better, 2% salicylic acid with 20% of sulphur in oxide of zinc ointment.

